# An Individualized Prognostic Signature for Clinically Predicting the Survival of Patients With Bladder Cancer

**DOI:** 10.3389/fgene.2022.837301

**Published:** 2022-03-29

**Authors:** Qing Liu, Yunchao Wang, Huayu Gao, Fahai Sun, Xuan Wang, Huawei Zhang, Jianning Wang

**Affiliations:** ^1^ Department of Medical Ultrasound, Shandong Provincial Hospital Affiliated to Shandong First Medical University, Jinan, China; ^2^ Department of Urology, The First Affiliated Hospital of Shandong First Medical University, Jinan, China; ^3^ Department of Urology, Fifth Peoples Hospital Jinan, Jinan, China

**Keywords:** bladder cancer, prognostic model, tumor immune microenvironment, bioinformatics analysis, immunologic gene

## Abstract

**Background:** The tumor immune microenvironment (TIME) plays an important role in the development and prognosis of bladder cancer. It is essential to conduct a risk model to explore the prognostic value of the immunologic genes and establish an individualized prognostic signature for predicting the survival of patients with bladder cancer.

**Method:** The differentially expressed immunologic genes (DEGs) are identified in The Cancer Genome Atlas (TCGA). The nonnegative matrix factorization (NMF) was used to stratify the DEGs in TCGA. We used the least absolute shrinkage and selection operator (LASSO) Cox regression and univariate Cox analysis to establish a prognostic risk model. A nomogram was used to establish an individualized prognostic signature for predicting survival. The potential pathways underlying the model were explored.

**Results:** A total of 1,018 DEGs were screened. All samples were divided into two clusters (C1 and C2) by NMF with different immune cell infiltration, and the C2 subtype had poor prognosis. We constructed a 15-gene prognostic risk model from TCGA cohort. The patients from the high-risk group had a poor overall survival rate compared with the low-risk group. Time-dependent ROC curves demonstrated good predictive ability of the signature (0.827, 0.802, and 0.812 for 1-, 3-, and 5-year survival, respectively). Univariate and multivariate Cox regression analyses showed that the immunologic prognostic risk model was an independent factor. The decision curve demonstrated a relatively good performance of the risk model and individualized prognostic signature, showing the best net benefit for 1-, 3-, and 5-year OS. Gene aggregation analysis showed that the high-risk group was mainly concentrated in tumorigenesis and migration and immune signaling pathways.

**Conclusion:** We established a risk model and an individualized prognostic signature, and these may be useful biomarkers for prognostic prediction of patients with bladder cancer.

## Introduction

Bladder cancer is the most common type of cancer among urinary malignancies and adds approximately 550,000 new cases each year, with approximately 200,000 deaths (Antoni et al., 2017; Bray et al., 2018). The traditional treatments for bladder cancer mainly include transurethral resection and cisplatin-based chemotherapy (Tran et al., 2021). However, the 5-year overall survival rate remains at 40–60% for muscle-invasive bladder cancer (Alfred Witjes et al., 2017). Immunotherapy, such as bacillus Calmette–Guérin (BCG) and immune checkpoint inhibitors (ICIs), has become effective and showed promising antitumor activity (Balar et al., 2021). Studies demonstrated that immunotherapy might benefit some patients with bladder cancer, suggesting that the tumor immune microenvironment (TIME) may play an important role (Boorjian et al., 2021; Witjes et al., 2021).

The TIME, representing a complicated network of suppressing cancer immunity (Binnewies et al., 2018), is essential for tumor cells to avoid potential autoimmunity and tissue damage while generating a successful immune escape and is involved in restraining tumor development or tumor-promoting effects and affects the response to immunotherapy (Chen et al., 2017; Lenis et al., 2020). This regulation is orchestrated by different mechanisms, either intrinsic or extrinsic. The TIME has been demonstrated to predict the prognosis for patients with different tumor types, including hepatocellular carcinoma (Gong et al., 2021), breast (Keren et al., 2018), and ovarian cancers (Jiang et al., 2020). Immune gene expression patterns are also enriched in UBC. Although several clinical features and molecular biomarkers have been applied for the prognosis of bladder cancer, only limited data of patient cohorts have indicated positive prognostic relevance of the TIME for patient survival. Therefore, it is meaningful for us to find out high accurate prognostic biomarkers, which could guide patient selection and help evaluate likely disease outcomes.

In this study, we used data from The Cancer Genome Atlas (TCGA) and GEO database to analyze the differential expression of immunologic genes and constructed a consistent clustering. Then, we conducted a risk model to explore the prognostic value of the differentially expressed immunologic genes and established an individualized prognostic signature for predicting the survival of patients with bladder cancer. We also characterized the signature based on the differentially expressed immunologic gene scores as a prognostic stratification tool.

## Materials and Methods

### Data Preparation

Gene expression data (FPKM format, normalized), related clinical information, and single-nucleotide variant (SNV) of the bladder cancer were obtained from The Cancer Genome Atlas (TCGA) database (https://portal.gdc.cancer.gov/). The data consisted of 414 bladder cancer samples and 19 normal case samples. RNA-seq data and clinical survival information of the GSE19423 dataset for additional bladder cancer samples were obtained from the Gene Expression Omnibus (GEO) (https://www.ncbi.nlm.nih.gov/geo/). GSE19423 had 48 tumor tissues with their associated follow-up information for subsequent validation of the prognostic gene signature. The samples with incomplete data on gender, age, survival time, survival status, and pathological grading were excluded. A total of 4061 immunologic gene sets (C7) were downloaded from Gene Set Enrichment Analysis (GSEA) (https://www.gsea-msigdb.org/gsea/msigdb/index.jsp) (Subramanian et al., 2005). The data were analyzed with the R (version 3.6.1) and R Bioconductor packages.

### Screening of the Differentially Expressed Immunologic Genes

Immunologic gene sets (C7), GSE19423 dataset, and TCGA bladder cancer datasets are used to extract and sort out the expression of immunologic genes. The differentially expressed immunologic genes between tumor and normal tissues in TCGA bladder cancer datasets were screened using edgeR package (Robinson et al., 2010), with parameters of |logFC| > 1.0 and *p*-value < 0.05. The differentially expressed immunologic genes were displayed by the volcano plot. (*n* = 1018)

### Bladder Cancer Subtype Identification

The nonnegative matrix factorization (NMF) was used to stratify the differentially expressed immunologic genes in TCGA. The “NMF” R package was used to perform unsupervised NMF clustering (Gaujoux and Seoighe, 2010). According to cophenetic, dispersion, and silhouette coefficients, the best cluster number was chosen as the coexistence correlation coefficient K value 2. To explore the profiles of two subtypes, we used Kaplan–Meier analysis (R package “survival”) to discriminate genes correlated with overall survival. Using the CIBERSORT algorithm (Newman et al., 2015), the infiltration levels of 23 kinds of immune cells were estimated, and we utilized the “MCPcounter” R package to evaluate the abundances of two stromal cells and eight immune cells in two subtypes (Becht et al., 2016).

### Construction of the Immunologic Prognostic Risk Model

To explore the prognostic value of the differentially expressed immunologic genes, we conducted a prognostic risk model. Before conducting the risk model, the randomly selected 70% samples from the differentially expressed immunologic genes were assigned as the training dataset, while the remaining 30% samples and GSE19423 dataset were used as the test set. The prognostic-related genes were identified by univariate and multivariate Cox regression analyses. After that, we conduct the least absolute shrinkage and selection operator (LASSO) Cox regression (R package “glmnet”) to identify independent prognostic genes powerfully associated (*p* < 0.05) with OS in bladder cancer patients (Friedman et al., 2010). The risk score was calculated by the following formula:

Risk score=∑(Coefficient of mRNAi∗exp⁡ression of mRNAi). (1)

Based on the calculating formula, the training and the test sets were divided into low- and high-risk groups based on the median risk score. To validate the model, the receiver operating characteristic curve (ROC) analysis (R package “timeROC”) and clinicopathological feature (age, gender, histological grade, and pathological T, N, and M) analysis (R package “survminer”, “survival”) were performed. Diagnosis of age, gender, histological grade, stage, and the risk score was included in this study for univariate and multivariate Cox regression analyses, which determined that the risk score was the independent prognostic factor for bladder cancer. We will also analyze which immunologic genes are related to the risk score. Then, we analyzed the relationship between the risk score and immune cell infiltration and tumor mutation burden (TMB) in bladder cancer through R package “corrplot” (Wei et al., 2017), and “circlize” (Gu et al., 2014).

### Construct and Validate the Individualized Prognostic Signature

A previous study showed that clinical characteristics, including age, gender, histological grade, stage, and pathological T, N, and M, were risk predictors for OS in bladder cancer (Shinohara et al., 1995; Puente et al., 2003; Gupta et al., 2009). Diagnosis of age, gender, histological grade, stage, pathological T, N, M, and the risk score was used to build an individualized prognostic signature (R package “rms”, “nomogramEx” and “regplot”) to predict OS (Du et al., 2017; Arnhold, 2018). Next, we estimated whether the predicted survival outcome (1-, 3-, and 5-year survival) was close to the actual outcome with calibration curves. Furthermore, 1-, 3-, and 5-year decision curve analysis (DCA), which can assess and compare prediction signatures that incorporate clinicopathological features, was used to calculate whether our established nomogram was suitable for clinical utility.

### Gene Set Enrichment Analyses and Identification of Significantly Mutated Genes

To identify the potential molecular mechanisms underlying the signature in the low-risk and high-risk groups, GSEA (Subramanian et al., 2005) was performed through the limma R package and clusterprofiler package. *p* < 0.05 was considered statistically significant.

### Statistical Analyses

*R* software 4.1.1 was applied in this study for statistical analyses. Categorical variables were analyzed *via* the Wilcoxon rank-sum test. Continuous variables were analyzed using Student’s t-test for paired samples. Univariate and multivariate Cox regression analyses were utilized to evaluate survival. The hazard ratio (HR) and 95% confidence interval (CI) were calculated to identify genes associated with OS. *p*-value < 0.05 was considered statistically significant.

## Results

### Identification of Differentially Expressed Immunologic Genes

**FIGURE 1:**
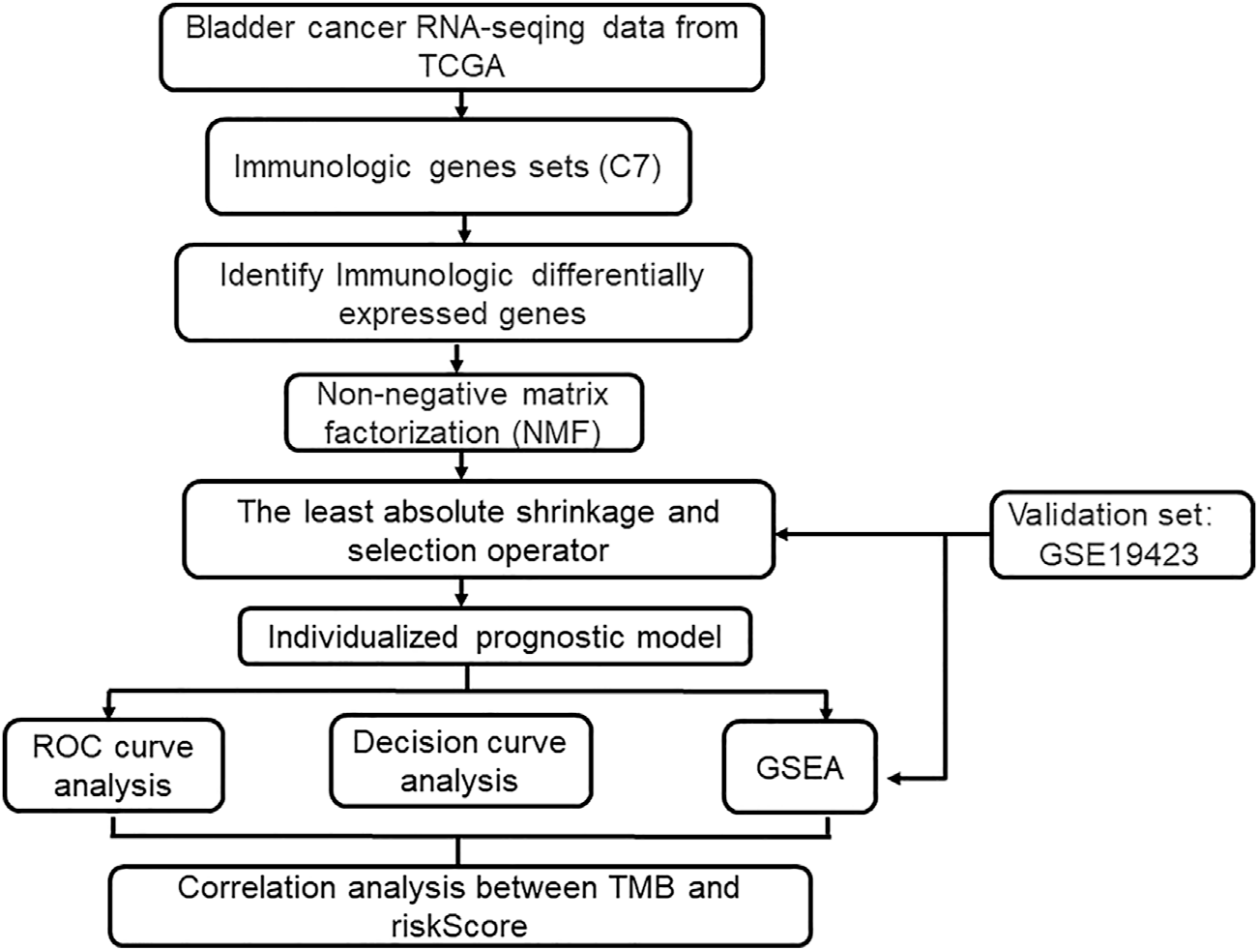
Flow chart of this study.

**FIGURE 2:**
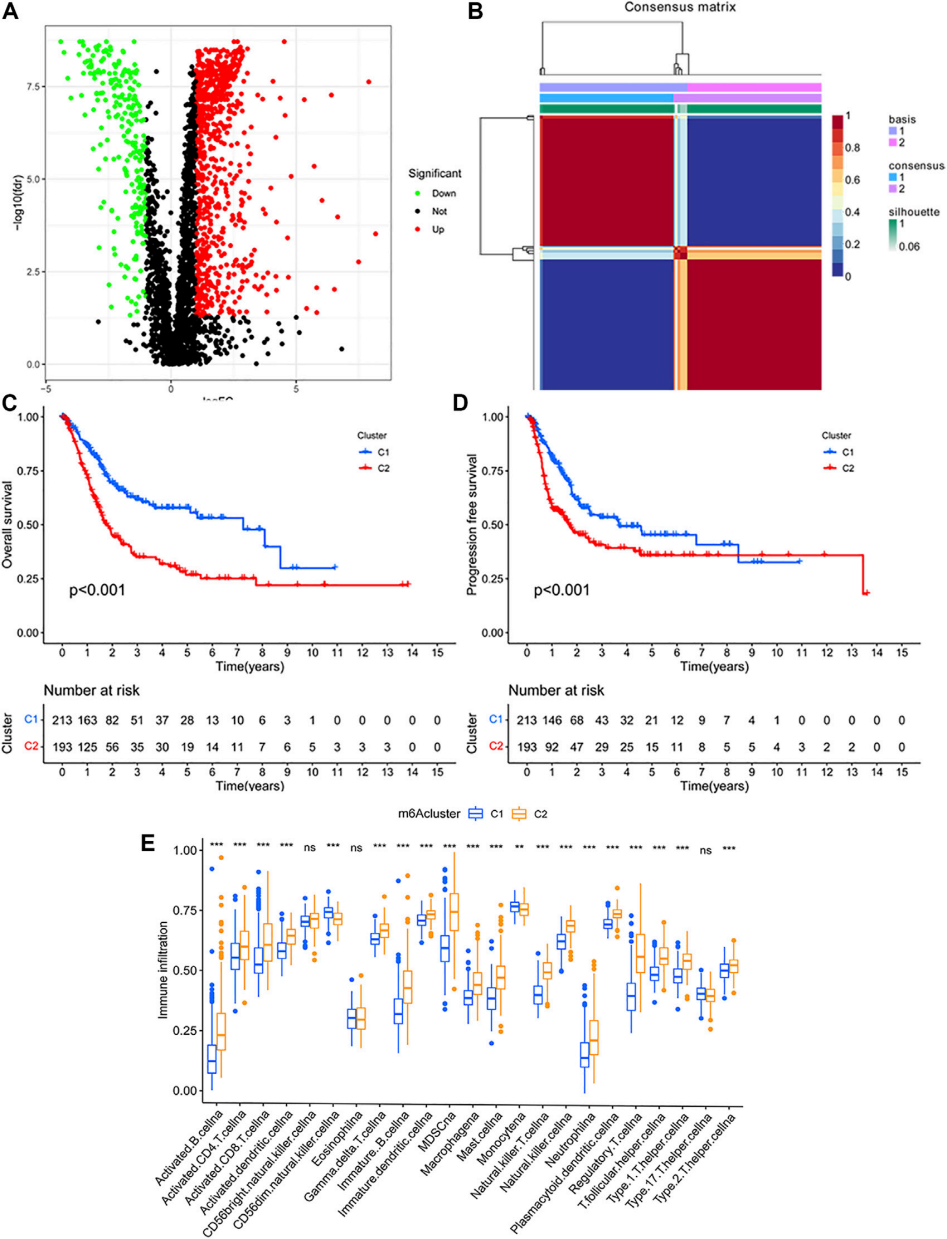
**(A)** Identification of the differentially expressed immunologic genes in TCGA database. **(B)** Identification of clinically relevant subtypes of bladder cancer: samples were clustered by the nonnegative matrix factorization (NMF) method. **(C,D)** Survival curves of each immune subtype in the training set. **(E)** Immune cell infiltration in two subtypes.

Our study flowchart is shown in Figure 1. 4061 immunologic gene sets were retrieved from “immunologic signature gene sets”. After the preliminary screening, we matched 1,018 DEGs between tumor and normal tissues in TCGA database (Supplementary Table S1). 773 genes were found to be significantly upregulated, while 245 genes were significantly downregulated (Figure 2A).

### Classification and Characterization of Bladder Cancer

Based on the expression profiles of the differentially expressed immunologic genes, bladder cancer samples from TCGA were clustered by using the NMF package. The optimal value of subtypes (K) was determined (K = 2, Figure 2B) on the grounds of the cophenetic correlation coefficient. Hence, the bladder cancer samples were clustered into two molecular subtypes C1 and C2. Bladder cancer patients with subtype 1 displayed good OS, while subtype 2 had poor prognosis (log-rank *p* < 0.001; Figure 2C). Interestingly, patients in subtype 2 had shorter PFS time than those in C1 (log-rank *p* < 0.001; Figure 2D).

To estimate the population abundance of tissue-infiltrating immune and stromal cell populations in two subtypes, we used the CIBERSORT algorithm and the MCPcounter method. As a result, activated B cell, activated CD4 T cell, activated CD8 T cell, activated dendritic cell, MDSC cell, macrophage, natural killer cell, type 1, 2 T helper cell, and regulatory T cell in cluster 2 were significantly higher than those in cluster 1 (*p* < 0.01), while monocyte (*p* < 0.05) and CD56 dim natural killer cell (*p* < 0.001) in cluster 1 were higher than those in cluster 2 (Figure 2E). The results from the MCPcounter method validated that given in Supplementary Figure S1.

### Construction of the Immunologic Prognostic Risk Model

**FIGURE 3:**
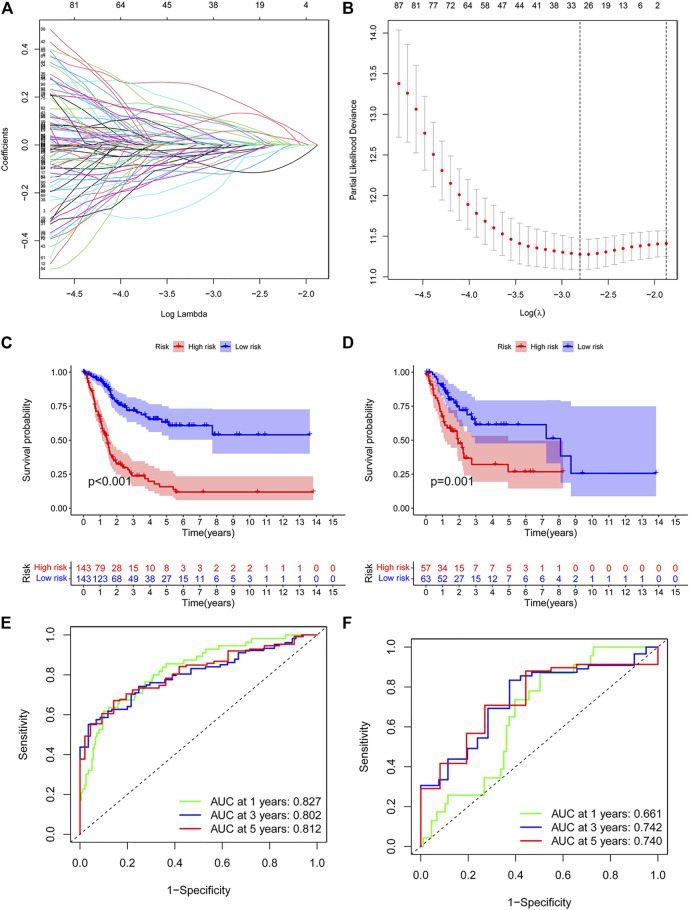
**(A,B)** Risk model as prognostic factors for bladder cancer patients. **(C,D)** Kaplan–Meier survival curves show the OS of patients in the high- and low-risk groups in the training group. **(E,F)** Time-dependent ROC curves illustrated the ability of the risk model to predict OS in the training and test set (training dataset: 1-year AUC = 0.827, 3-year AUC = 0.802, 5-year AUC = 0.812; test dataset: 1-year AUC = 0.661, 3-year AUC = 0.742, 5-year AUC = 0.740).

Then, we conducted a risk model to explore the value of differentially expressed immunologic genes. Among TCGA training cohort, 144 OS associated differentially expressed immunologic genes were identified through the univariate Cox regression analysis (*p* < 0.05) (Supplementary Table S2). In addition, the multivariate Cox regression analysis identified 15 OS associated differentially expressed immunologic genes in bladder cancer patients (*p* < 0.05) (Supplementary Table S3). Fifteen genes were screened using the LASSO regression algorithm (Figures 3A,B) to construct the immunologic prognostic risk model. Using the coefficients obtained from the expression levels and regression coefficients, patients from the training and the test set were divided into low-risk and high-risk groups based on the median risks score to assess the robustness of the prognostic risk model. The patients from the high-risk group had a poor overall survival rate compared with those in the low-risk group (Figures 3C,D) whether in the training or test set. Time-dependent ROC curves showed that the classifier had good accuracy with 0.827, 0.802, and 0.812 for 1-, 3-, and 5-year survival, respectively, in the training set while 0.661,0.742,0.740 in the test set. (Figures 3E,F). Moreover, the immunologic prognostic risk model had better predictive power and accuracy than other clinical factors (including age, gender, grade, and stage), indicating an independent prognostic risk factor (Supplementary Figure S2).

**FIGURE 4:**
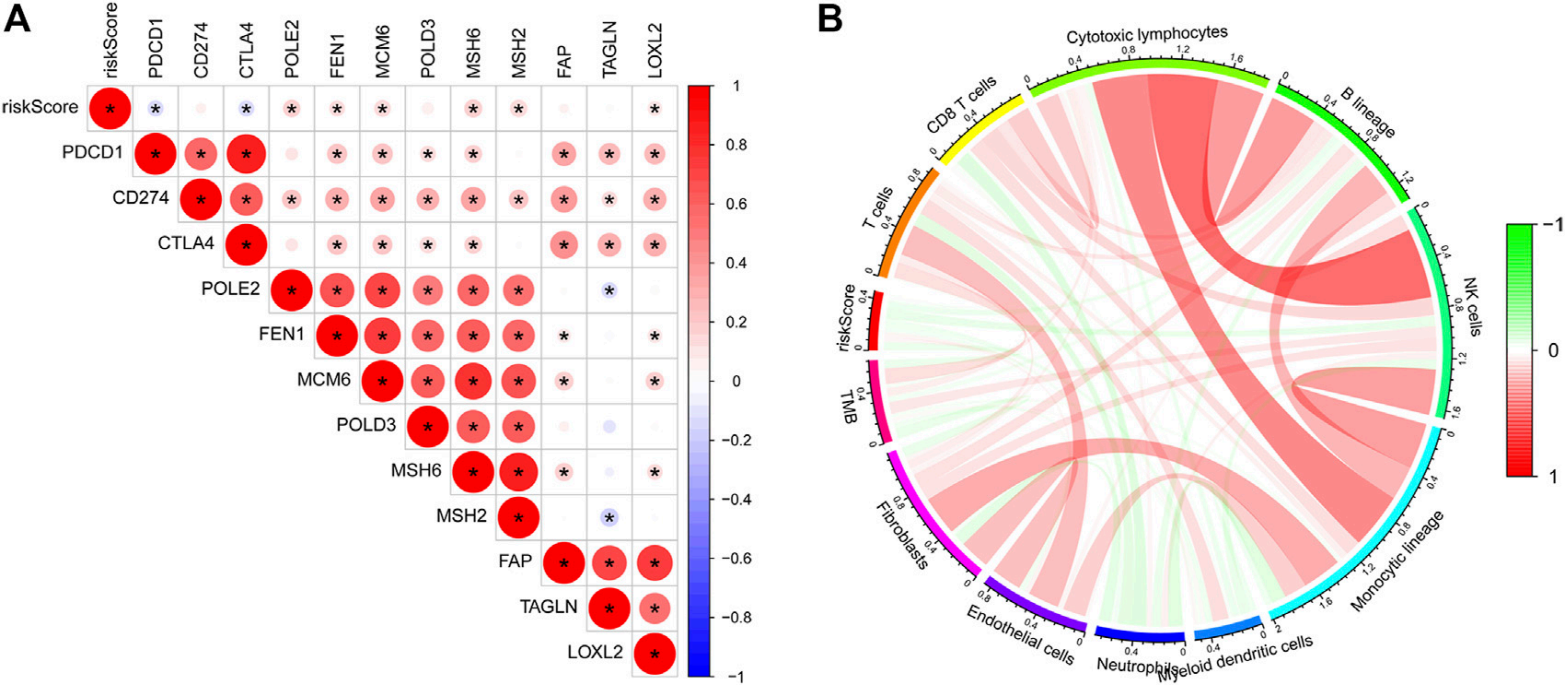
**(A)** Correlations between the risk score and immunologic genes: a negative correlation was marked with blue and a positive correlation with red. **(B)** Correlation between the risk score and TMB and immune cells: a negative correlation was marked with blue and a positive correlation with red.

According to the analysis between immunologic genes and the risk score, we found that POLE2, FEN1, MCM6, MSH6, MSH2, and LOXL2 are positively correlated with the risk score, while PDCD1 and CTLA4 are negatively correlated with the risk score (Figure 4A). We also analyze the relationship between the risk score and immune cell infiltration and TMB (Figure 4B). The results showed that the risk score and TMB have a negative correlation and the risk score and endothelial cells have a positive correlation. In addition, we can also observe that except for endothelial cells, most immune cells have a negative relationship with the risk score. Univariate and multivariate Cox regression analyses showed that the immunologic prognostic risk model was an independent factor (Table 1).

**TABLE 1:** Cox analysis of clinicopathological parameters for OS in bladder cancer.

	Univariate			Multivariate		
	HR	95% CI	*p*	HR	95% CI	*p*
Age	1.03	1.02–1.05	0.00002	1.02	1.00–1.03	0.005
Stage	1.74	1.44–2.11	0.00000002	1.59	1.30–1.93	0.000004
RiskScore	1.11	1.09–1.13	0.005^*^	1.08	1.06–1.10	0.005^*^

^*^Statistically significant (*p* < 0.05).

We used the GSE19423 cohort to validate the predictive ability of the immunologic prognostic risk model. Kaplan–Meier analysis showed that the low-risk group has a greater chance of obtaining the same survival time than the high-risk score group in the GEO dataset (Supplementary Figure S3).

### Establishment of an Individualized Prognostic Signature

**FIGURE 5:**
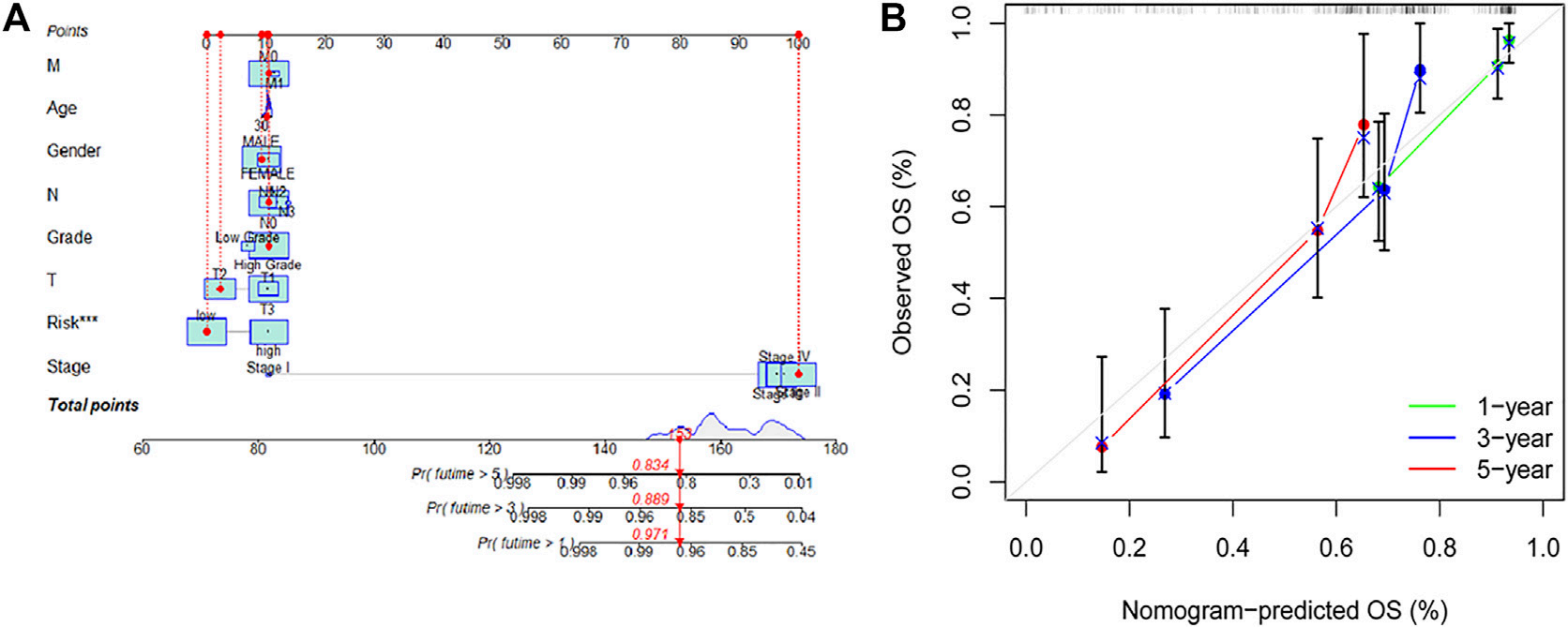
An individualized prognostic signature incorporating the risk score and clinical factors for bladder cancer prognosis. **(A,B)** Construction of an individualized prognostic signature combining the subtype-specific signature and clinical features for prediction of OS. Calibration plots displayed the actual and signature-predicted probability of 1-, 3- and 5-year OS.

Based on the results of the univariate and multivariate analyses, diagnosis of age, stage, and risk score were independent risk predictors for overall survival (OS) (Supplementary Table S4). An individualized prognostic signature was generated to observe the relationship between these independent prognostic factors and personalized survival status (Figure 5A). The calibration curve was constructed to describe the prediction value of the nomogram, and the 45-degree line indicated the actual survival outcomes (Figure 5B). In the calibration curve, the signature had good predictive power for predicting OS at 1, 3, and 5 years.

**FIGURE 6:**
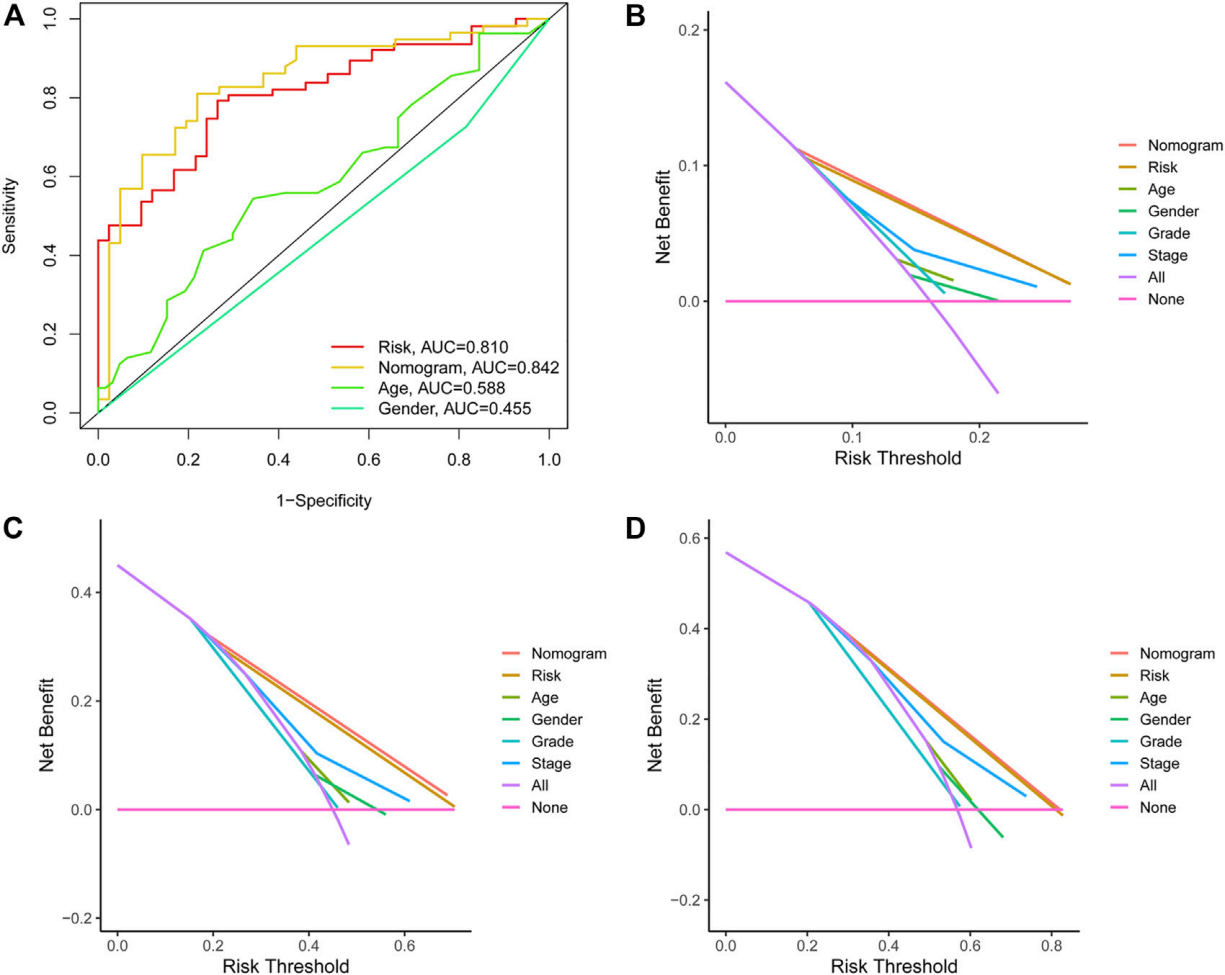
**(A)** ROC curves illustrated the ability of the risk model and individualized prognostic signature to predict OS in the training set. **(B–D)** Decision curve analysis of the individualized prognostic signature and riskScore for the survival prediction of patients in 1-,3-, and 5-year survival benefit in the training cohort.

In addition, the ROC analysis result demonstrated that the AUC of individualized prognostic signature and nomogram signature increased to 0.791 and 0.784, respectively (Figure 6A). The DCA was performed for the clinicopathological features (including age, gender, grade, and stage), risk signature, and nomogram signature as shown in Figure 6B. The decision curve demonstrated relatively good performance of risk signature and individualized prognostic signature, showing the best net benefit for 1-, 3-, and 5-year OS (Figures 6C,D).

### Functional Analysis of the Prognostic Signature

To understand the biological processes and KEGG pathways of the obtained 15 OS-associated differentially expressed immunologic genes, gene set enrichment analysis was performed (Figures 6A,B). As shown in Figure 6A, five biological processes relevant to ECM receptor interaction, focal adhesion, JAK-STAT, pathway in cancer, and regulation of actin cytoskeleton were enriched in the high-risk group. These results manifest that the functions of these genes are mainly embodied in the regulation of information transmission between cells and the transfer of nanoparticles and tumorigenesis.

## Discussion

**FIGURE 7:**
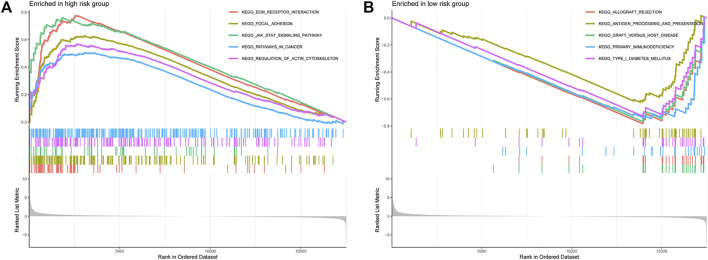
**(A,B)** KEGG enrichment analysis of the high- and low-risk groups.

Bladder cancer is one of the most prevalent cancers worldwide, with ∼430,000 new diagnoses each year (Antoni et al., 2017). Although few advances have improved the clinical management of bladder cancer over the past 20 years, the overall incidence and mortality rates have changed little (Kamat et al., 2015). Immunotherapy has been used to treat bladder cancer for more than 40 years by using BCG. BCG was highly effective against bladder tumors and has been a part of the standard treatment for patients with bladder cancer. Cisplatin-ineligible patients or patients with metastatic urothelial carcinoma have limited treatment options. The ICIs have led to the advent of new classes of drugs, demonstrating promising results and improved response (Rosenberg et al., 2016). ICIs have become one of the most promising areas in cancer therapeutic development, but still not every patient achieves clinical benefit. Bladder cancer is one of the most significant genitourinary cancers with a high rate of somatic mutations. The TME has a critical influence on the immune response. In the TIME, there were distinct differences in immune cell infiltrations between subtypes. Tumor-infiltrating immune cells are linked to clinical outcomes and response to immunotherapy. Constructing reliable signatures to assess the clinicopathological features and analyze immune cell infiltration in bladder cancer patients is essential. Figure 7.

In this study, we identified the differentially expressed immunologic genes and divided bladder cancer samples into two subtypes by NMF cluster analysis. It was found that patients in subtype 1 had longer mPFS and mOS than those in C2. As we can see, cluster 2 contained more regulatory T cells (Treg), and myeloid-derived suppressor cells (MDSCs), which have a negative immunomodulatory effect (Siret et al., 2020). Our study focused on bioinformatics to predict the diagnostic, therapeutic, and prognostic value of immunologic genes in bladder cancer. Then, an immunologic prognostic risk model and individualized prognostic signature were constructed. We also identified 15 OS-related differentially expressed immunologic genes, and 10 genes (e.g., TSPAN7, PAQR6, TRIM59, RUNX2, AIM2, CGB5, FASN, FADS1, RAC3, and HLA-G′) (Prasad et al., 2013; Liu et al., 2018; Dumont et al., 2019; Cheng et al., 2020; Jiao et al., 2020; Liu et al., 2020; Piotr Białas et al., 2020; Cai et al., 2021; Yu et al., 2021) have been reported to be involved in the immune microenvironment. POLE2, FEN1, MCM6, MSH6, MSH2, and LOXL2 were considered to play a role in promoting cancer. A whole-genome CRISPR screen study found that MSH2 was involved in chemotherapy resistance in muscle-invasive bladder cancer and might be predictive biomarkers of response to platinum-based therapy (Goodspeed et al., 2019). The relationship between TMB and the risk score has also been explored, and it is found that the TMB and risk score have a negative correlation. TMB is defined as the total number of somatic mutations per megabase of an interrogated genomic sequence, and high TMB may produce many neoantigens to stimulate the antitumor immune response (Chan et al., 2019). In this study, we observe that endothelial cells have a positive relationship with the risk score. Studies showed that bladder cancer cells promoted tumor progression interacting with vascular endothelial cells through the VEGFR2 and EGFR signaling pathway (Huang et al., 2019). The differences in age, stage, gender, and grade did not reduce the accuracy of the classifier in predicting patient prognoses (Supplementary Figure S2). The nomogram incorporates an immunologic prognostic risk signature and clinicopathological parameters to help clinicians determine individual patient prognoses. Its graphical scoring system is easy to understand, facilitating the customized treatment and making of medical decisions. The ROC curves indicated that the immunologic prognostic risk signature and prognostic nomogram signature had high sensitivity and specificity. The results of the calibration curve and DCA demonstrated that the two signatures could be independent factors affecting the prognosis of bladder cancer and may be practical and reliable predictive tools for predicting bladder cancer prognosis.

In this study, we conducted GSEA to screen the most important signaling pathways. The results of GSEA indicated that the high-risk group was mainly concentrated in tumorigenesis and migration and immune signaling pathways, suggesting that bladder cancer in the high-risk group had a higher level of cell proliferation and immunosuppression.

Previous studies have reported different prognostic signatures of bladder cancer (Na et al., 2020; Qiu et al., 2020; Fu et al., 2021). However, most of them focused on building prognostic models. In this study, we comprehensively evaluated the prognostic value of immunologic genes in bladder cancer, identified two bladder cancer subtypes by unsupervised NMF clustering, and more importantly, established an individualized prognostic signature for predicting the survival of patients with bladder cancer. In this study, we also analyzed the relationship between the risk score and immune cell infiltration and TMB. It provides a new perspective for improving the response of bladder cancer to immunotherapy. Some limitations of this study should be noted. First, the study relied on retrospective data, so there was a lack of verification by multicenter prospective research. Second, the nomogram did not perform external validation as there was a lack of specific clinical data in the GEO database.

In conclusion, we established a risk model and an individualized prognostic signature, and this signature is more economical and clinically practical than whole-genome sequencing. The graphical scoring system of our nomogram is easy to understand, facilitating the customized treatment and making of medical decisions. Furthermore, this signature could be used to guide clinicians in decisions related to prognosis, clinical diagnosis, and medication for bladder cancer patients with different immunophenotypes. Understanding the TIME using the immune score provides important insights that will improve the diagnosis and prognosis of patients with bladder cancer.

## Data Availability

The original contributions presented in the study are included in the article/Supplementary Material, further inquiries can be directed to the corresponding authors.

## Abbreviations

AUC, the area under the curve; BCG, bacillus Calmette–Guérin; CI, confidence interval; DCA, decision curve analysis; FDR, false discovery rate; GEO, Gene Expression Omnibus; GSEA, Gene Set Enrichment Analysis; HR, hazard ratio; ICIs, immune checkpoint inhibitors; LASSO, least absolute shrinkage and selection operator; MDSCs, myeloid-derived suppressor cells; OS, overall survival; PFS, progression-free survival; ROC, receiver operating characteristic curve; SNV, single-nucleotide variant; TCGA, The Cancer Genome Atlas; TIME, tumor immune microenvironment; TMB, tumor mutation burden; Treg, regulatory T cells.
