# Supplementary material for: An Individualized Prognostic Signature for Clinically Predicting the Survival of Patients With Bladder Cancer
Source: Front Genet. 2022 Mar 29;13:837301. doi: 10.3389/fgene.2022.837301 (PMC9002098; doi:10.3389/fgene.2022.837301)
Supplement: Supplementary file 4 [file Presentation1.PPTX]

## Slide 1
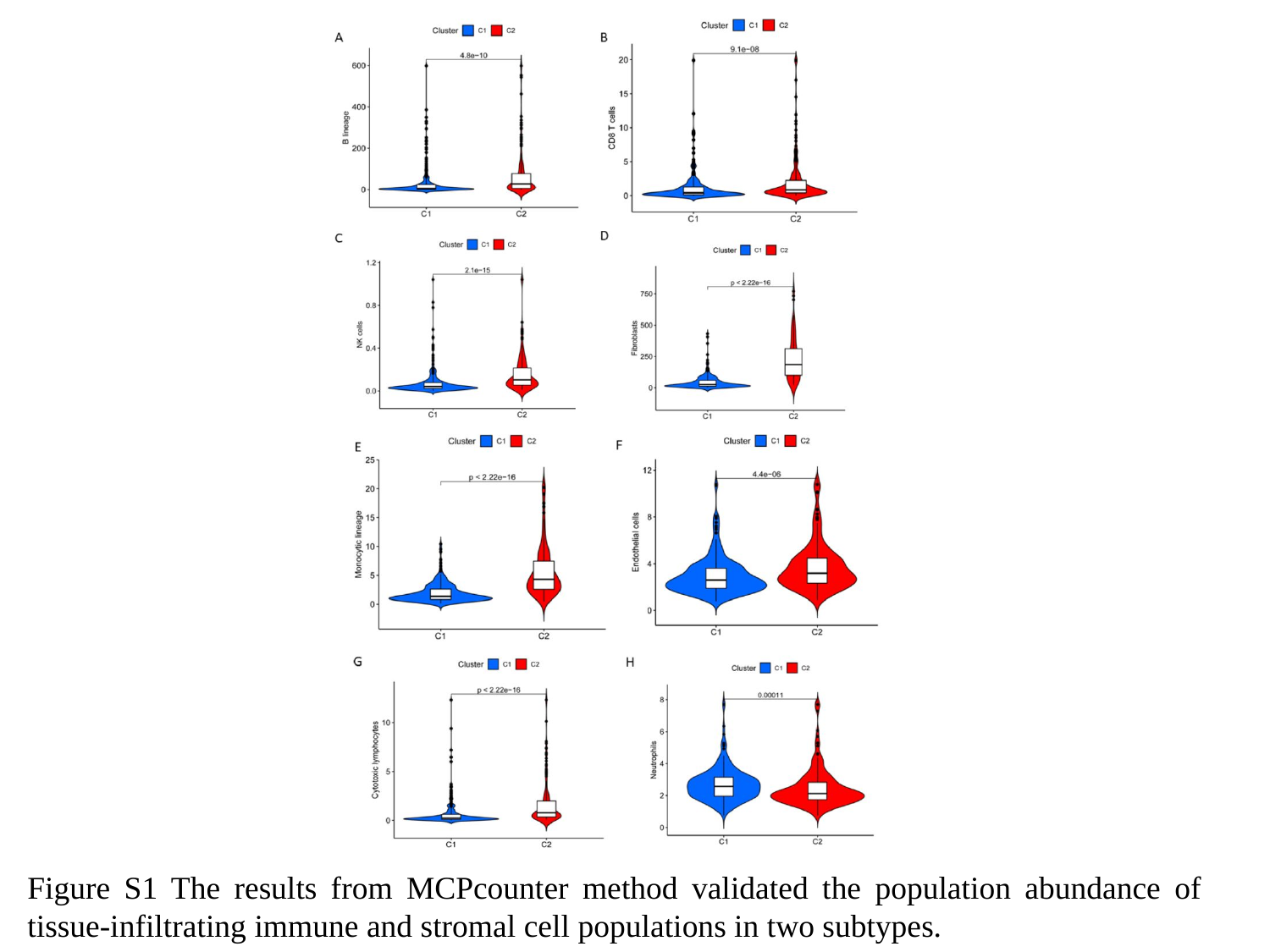

Figure S1 The results from MCPcounter method validated the population abundance of tissue-infiltrating immune and stromal cell populations in two subtypes.

## Slide 2
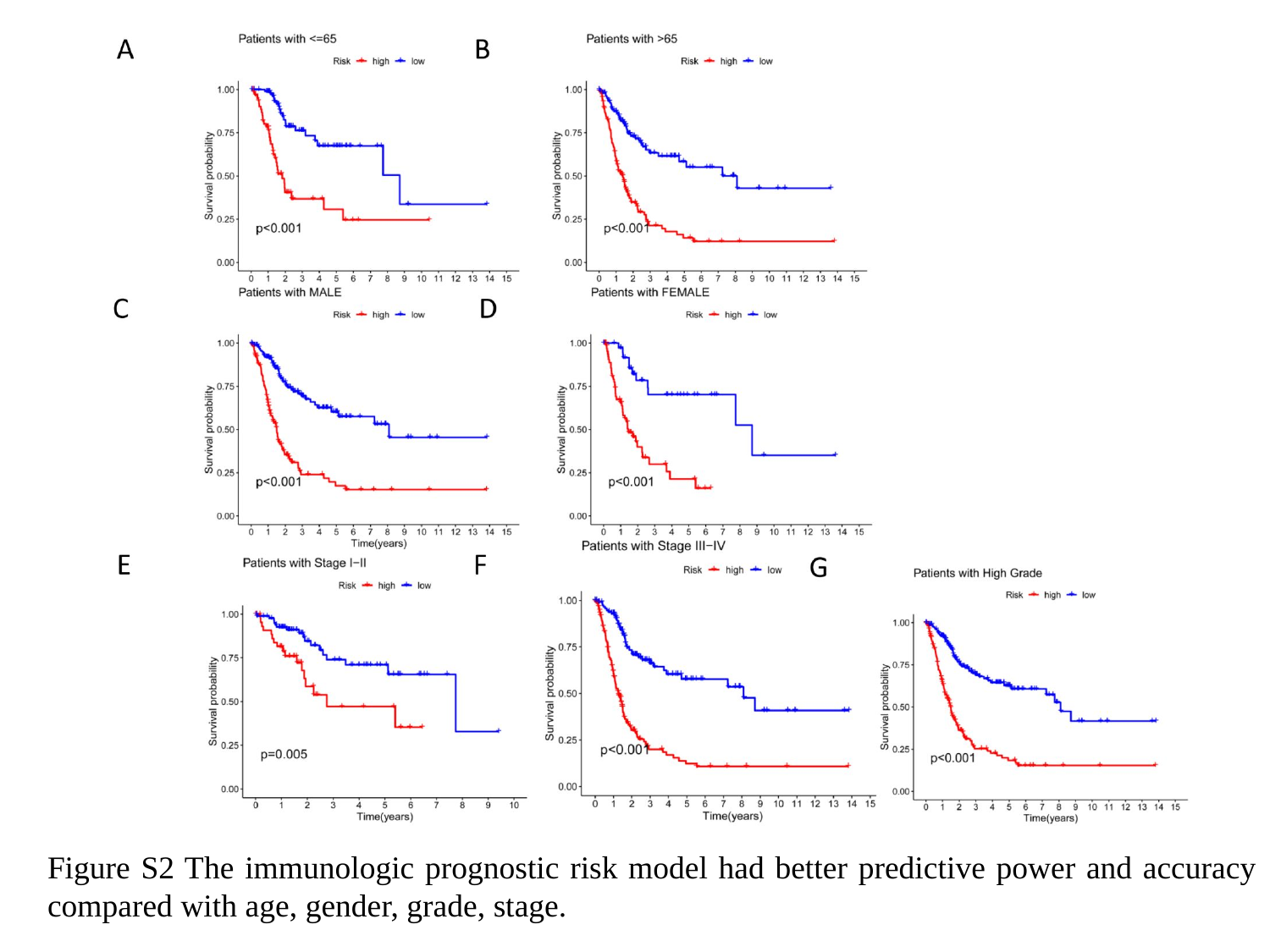

Figure S2 The immunologic prognostic risk model had better predictive power and accuracy compared with age, gender, grade, stage.

## Slide 3
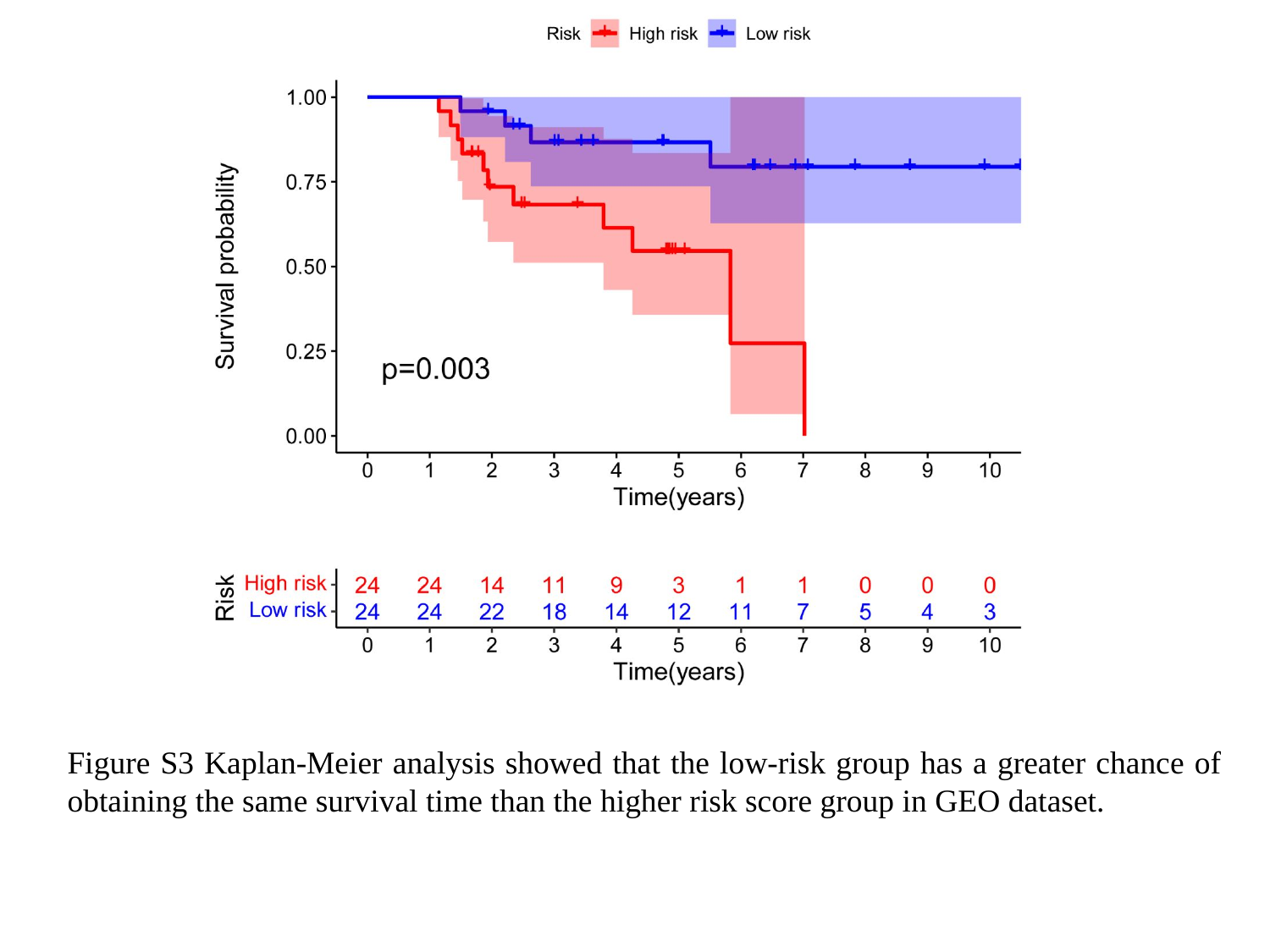

Figure S3 Kaplan-Meier analysis showed that the low-risk group has a greater chance of obtaining the same survival time than the higher risk score group in GEO dataset.
